# Parents Working Together: development and feasibility trial of a workplace-based program for parents that incorporates general parenting and health behaviour messages

**DOI:** 10.1186/s12889-016-3817-z

**Published:** 2016-11-10

**Authors:** L. Wilson, Donna Lero, Allan Smofsky, Deborah Gross, Jess Haines

**Affiliations:** 1 Department of Family Relations and Applied Nutrition, University of Guelph, Guelph, ON Canada; 2 Managing Director of Smofsky Strategic Planning, Oakville, ON Canada; 3 School of Nursing, Johns Hopkins University, Baltimore, MD USA

**Keywords:** Childhood obesity prevention, Parenting program, Feasibility trial, Workplace, Health behaviours

## Abstract

**Background:**

Parenting programs integrating general parenting and health behaviour messaging may be an effective childhood obesity prevention strategy. The current study explored workplaces as an alternate setting to deliver parenting programs.

**Methods:**

This study involved two phases. The objective of the first phase was to explore interest in and preferred delivery mode of a workplace program that addresses general parenting and health behaviours. The objective of the second phase was to adapt and test the feasibility and acceptability of a pre-existing program that has been successfully run in community settings for parents in their workplace. To achieve the first objective, we conducted 9 individual or small group qualitative interviews with 11 workplace representatives involved in employee wellness/wellness programming from 8 different organizations across Southwestern Ontario. To achieve the second objective, we adapted a pre-existing program incorporating workplace representatives’ suggestions to create Parents Working Together (PWT). We then tested the program using a pre/post uncontrolled feasibility trial with 9 employees of a large manufacturing company located in Guelph, Ontario.

**Results:**

Results from the qualitative phase showed that a workplace parenting program that addresses general parenting and health behaviour messages is of interest to workplaces. Results from the feasibility trial suggest that PWT is feasible and well received by participants; attendance rates were high with 89 % of the participants attending 5 or more sessions and 44 % attending all 7 sessions offered. All participants stated they would recommend the program to co-workers. Just over half of our parent participants were male (55.6 %), which is a unique finding as the majority of existing parenting programs engage primarily mothers. Impact evaluation results suggest that changes in children’s and parents’ weight-related behaviours, as well as parents’ reports of family interfering with work were in the desired direction post-intervention; however, confidence intervals substantially overlapped zero. Contrary to expectations, parents also reported an increase in restrictive feeding practices.

**Conclusion:**

Our results indicate that a workplace-based program that addresses general parenting skills and weight-related behaviours may be a feasible way to engage and educate parents, including fathers. A full-scale trial is needed to examine the effectiveness of this approach.

**Electronic supplementary material:**

The online version of this article (doi:10.1186/s12889-016-3817-z) contains supplementary material, which is available to authorized users.

## Background

In Canada, an estimated 19.8 % of children ages 5–17 years are overweight and an additional 11.7 % are obese [1]. Canadian preschool-aged children show similar trends with approximately 30 % of children between the ages of 2–5 years are overweight or obese [2]. Obesity in childhood is associated with adverse health effects for the affected child including hypertension, dyslipidemia, hyperinsulinemia/insulin resistance [3] and other risk factors associated with metabolic syndrome [4]. It is also well established that childhood overweight and obesity persists into adulthood and is associated with a host of comorbidities in later life [3, 5, 6].

The preschool years are a critical time for obesity prevention interventions, as habits developed in childhood tend to persist into later life [7–9]. At this age, parents are the primary influence in their children’s lives. They are responsible for the environment in which children are raised [10]. They also shape their children’s weight-related behaviours through specific feeding practices [10–12] and role modeling of activity and eating behaviours [13, 14]. In addition to these weight-specific parenting influences, general parenting style and practices, such as limit setting, have also been shown to be associated with obesity risk in children [15–17]. Integrating messaging related to general parenting and weight-related behaviours into parenting programs has been identified as a potentially effective obesity prevention strategy [18–20]. Parents and Tots Together (PTT) is one such obesity prevention intervention [18]. PTT is guided by the social contextual framework and embeds weight-related behaviour messaging within a general parenting program to be delivered to parents of children ages 2–5 years [18]. Results from a feasibility trial of PTT in community-based settings in Canada show that PTT was well received by parents (100 % of participants reported being either satisfied or very satisfied with the program) [19]. Additionally, compared to those in the control, parents who received the PTT intervention reported using food as a reward less frequently at post-intervention (β = -0.50, 95 % CI -0.90, -0.11, *p* = 0.01). Parents in the intervention also reported less parental stress (β = -20.67, 95 % CI -31.67, -9.62, *p* = 0.001) and greater confidence in managing children’s behaviour (β = 0.32, 95 % CI 0.04, 0.61, *p* = 0.03) compared to those in the control arm [19]. Other interventions using this approach have also found improvements in parental feeding practices [20, 22], as well as general parenting [21, 23]. Parenting programs addressing weight-related behaviours have typically been run in community-based settings (such as preschools, child care centres or health centres) and high rates of attrition and low attendance are common challenges of these community-based parenting programs [20, 21, 23, 24], suggesting the need to identify alternative approaches to reach parents of preschool-aged children.

As demographic trends change and an increasing proportion of families are dual earners (i.e., both parents work outside the home) [25], workplaces present a potentially effective setting to reach parents for programs focused on childhood obesity prevention. To date, only a small number of studies have examined the effectiveness of workplaces as a setting for parenting programs on a variety of topics including general parenting, sexual health and HIV prevention, and substance abuse, and the results have been positive with good attendance and improvements in the targeted behaviours [26–32]. To our knowledge, no obesity prevention interventions integrating general parenting and child weight-related messaging have been tested in the workplace setting. In this article, we describe the process of adapting Parents and Tots Together to the workplace setting and present the results of a feasibility trial of our workplace parenting program (Parents Working Together - PWT) at a single worksite.

In order to maximize the feasibility and acceptability of the intervention, we utilized a mixed methods approach with a sequential exploratory design [33]. The first step in this study was to gain an understanding from employers of their interest in and preferred delivery mode for a workplace parenting program. As such, the first section of this article will present our formative assessment, which involved qualitative methods - a series of individual and small group interviews with 11 representatives from a variety of workplaces across Southwestern Ontario. Knowing that workplaces may have certain logistical constraints (e.g., fixed lunch and break times), the goals of the formative assessment were to identify: a) employers’ perceived need for a program such as PWT in the workplace; b) logistical aspects of how a parenting program could be successfully delivered in the workplace; and c) workplace metrics to be added to the evaluation protocol that would be of interest to employers.

In the second section of this article we describe results from a pre/post uncontrolled feasibility trial of the PWT intervention – the quantitative piece to our mixed methods design. The primary objective was to determine the achievability and acceptability of PWT to employees and the secondary objective was to explore the extent to which the intervention was associated with changes in: a) child and parent weight-related behaviours (i.e., intake of sugar sweetened beverages, television viewing, sleep habits, physical activity); b) general parenting, feeding behaviours, parenting efficacy, and general stress; and c) measures of work-life balance.

## Methods: formative study

### Study design and participants

From April-July 2014 we conducted a series of individual or small group interviews (*n* = 2) with workplace representatives involved in employee wellness/wellness programming, (e.g., human resource personnel and occupational health nurses), from 8 workplaces. Criteria used to select these workplaces included: 1) located in Southwestern Ontario; 2) had a workplace representative able to speak English. The workplace representatives did not need to currently be offering a parenting or other workplace wellness program to be eligible to participate. A member from our research team who is a human resource consultant (AS) identified 11 potential representatives from workplaces that met the inclusion criteria. All identified workplace representatives were sent a scripted email to determine their interest in participation along with a one-page document outlining the rationale for the study. All 11 workplace representatives who were contacted agreed to participate in the study. Of the 11 workplace representatives interviewed, all were female, 8 were human resources (HR) personnel and 3 were occupational health nurses.

### Data collection

The semi-structured interview guide was developed by the research team to include questions relating to a) the perceived need for a program such as PWT in the workplace, b) logistical aspects of how a parenting program could be successfully delivered in the workplace, and c) workplace metrics to be added to the evaluation protocol that would be of interest to employers.

The interviews were held at the workplaces of the representatives being interviewed during daytime working hours. A member of the research team (LW), who has prior experience with conducting qualitative interviews and focus groups, moderated the interviews. An additional member of the research team (AS) was present to take notes. All the interviews were audio recorded and transcribed. Theoretical saturation was reached by consensus (AS, JH, LW) after which data collection ended.

### Data analysis

Transcripts were organized for analysis through Microsoft Excel tables and thematic analysis was used to analyze the transcripts [33]. A deductive or directive approach was used whereby the questions in the interview guide served as the framework for devising the initial codes [34–36]. One member of the research team (LW) reviewed the transcripts and generated a list of initial thematic codes using the questions from the interview guide for structure. Any additional data that did not seem to fit within the codes determined by the interview guide were coded separately and new categories were identified. A second researcher, not involved in the data collection, was then brought in to independently review the transcripts and organize the data into the coding schema. The two researchers met to ensure concordance in the coding. There was no instance where concordance could not be reached between the two researchers. Together, the researchers defined and named the categories and a report was generated summarizing the findings from the qualitative interviews as well as selecting the quotes that best illustrated the emergent categories to be used for member checking with interview participants. Responses were received from all 11 participants and there were no significant edits to be made as the participants felt that the summary provided to them accurately reflected their comments/thoughts from the interviews.

## Results: formative study

Six main categories of data were identified; five of the categories were predetermined by the interview guide (perceived need for program, existing or past program strategies used by employers, barriers to program implementation, suggested program logistics and strategies, and ways to address them, and suggested program evaluation) while one additional category emerged throughout the discussions with participants (suggestions for marketing the program). See Additional file 1 for representative quotes from each category. In the following sections we further describe findings related to each category.

### Perceived need for the program: work-life balance issues exist for employees and there is interest in a workplace parenting program

Universally, participants identified that issues with work-life balance are a significant problem for their employees. Many workplace representatives felt that employees’ struggles with work-life balance have been increasing in recent years and this was seen as leading to significant stress and mental health issues for employees, and employers are seeing this impacting workplace performance (i.e., presenteeism, absenteeism, productivity, decreases in engagement scores, and increases in health-related benefits costs). In addition to affecting workplace performance, participants identified that the stress resulting from struggles with work-life balance also impacts the personal lives of employees.

Overall, when asked about their interest in a workplace parenting program as a tool to ease the stress of work-life balance, most participants were very supportive of such an initiative. One participant even suggested that this is a program worth piloting.

### Existing or past program strategies used by employers

When asked about existing or previous programs available to support employee wellness, the majority of participants stated their organizations currently have initiatives to try to support employee wellness; however, none currently offer on-site parenting support programs. Participants reported that their organizations were currently using a number of strategies to address employees’ work-life balance issues (e.g., a supportive culture, flexible work hours/shifts, modified return to work policies after parental and other leaves, workshops, lunch-and-learns). The most common being referral to Employee Assistance Programs’ services, which provide counselling to employees on a broad range of mental health topics, as well as referrals to community and other resources as required.

### Barriers to program implementation and ways to address them

When asked about the potential barriers to implementing a parenting program in the workplace, participants identified four principal barriers that may arise: poorly attended sessions, stigma associated with attending a parenting program, lack of time, and other competing priorities. Participants felt that in their experience, lunch-and-learns tend to be poorly attended and that a way to maximize attendance was to offer incentives to attend (e.g. provide lunch, offer prizes). In order to reduce the stigma that may be associated with attending a parenting program held in the workplace, participants suggested marketing the program in a positive way (e.g. a “healthy families” program) versus as a program for families who are struggling. Finally, to address the issues of lack of time and other competing priorities, participants suggested a couple of strategies: showing the relevance to employees and employers so that a parenting program becomes a priority and having structured and focused sessions that are clearly communicated to employees.

### Suggested program logistics and strategies

Common suggestions to ensure the program is feasible for delivery in the workplace setting included: keeping the number of program sessions offered to the minimum required, holding sessions in the workplace during workday hours, using a traditional lunch and learn format, and shortening program sessions to fit into the employees’ allotted lunch break.

### Suggested program evaluation

When asked about program evaluation, all participants felt that conducting an evaluation was critical and almost all of the participants mentioned employee engagement as being an important metric to measure. Other measures of interest that were mentioned included financial return on investment and employee satisfaction with the program.

### Suggestions for marketing the program

Most suggestions for marketing the program to employees centred on using positive messaging (e.g. a “healthy families program” as mentioned above as a way to avoid stigma). Another suggestion was to focus on parenting struggles as a normal part of raising children. Participants also suggested focusing on the potential benefits associated with the participating in the program, specifically the reduced stress associated with improved parenting. In order to get employer buy-in for such a program, participants discussed stressing that a workplace parenting program could be a solution to work-life balance struggles faced by employees.

#### Key learnings from the formative assessment & implications for program adaptation

There were a number of key learnings from our formative assessment with workplace representatives that informed the adaptation of PWT – our workplace parenting program (Table 1).

**Table 1:** Overall findings from the formative assessment with employers

Employees are currently struggling with work-life balance issues and a worksite parenting program is worth piloting in a workplace setting.	
Deliver program in a lunch-and-learn format; however, shorten sessions to 30 min in length.	
Create positive messaging to market the program to employees.	
Create structured and focused sessions that are clearly communicated to employees.	
Expand the evaluation protocol to include measures of work-life balance and employee engagement.	

Taking these learnings, PWT sessions were condensed from 1.5 h to 30 min in length to fit into the employees’ lunch time and the program was shortened from 9 to 7 weeks in length. Additionally, the eligibility criteria were expanded so that the program could be offered to parents of children age 2–7 years (rather than 2–5 years used in previous trials of PTT). Given that the content of PWT is specific to the early years, the eligibility criteria could not be expanded further, but an effort was made to maximize the portion of employees that would be eligible to participate.

Second, employers suggested that there are a number of potential barriers to attending a parenting program held in the workplace (poorly attended sessions, stigma associated with attending a parenting program, and time and other competing priorities) and they had suggestions for increasing employee buy-in and maximizing attendance, including: positive marketing of the program and structured focused sessions that are clearly communicated to employees so that employees are aware of what is involved in the program prior to signing up.

Finally, our results suggest that an evaluation of the program is an important component in order to get organizational support for PWT. If employers are to support a workplace program they are interested in seeing workplace-related outcomes achieved by their employees. To capitalize on employer enthusiasm, the evaluation protocol for PWT was expanded to include measures of work-life balance and employee engagement.

## Methods: feasibility trial of parents working together (PWT)

### Study design and participants

**Figure 1:**
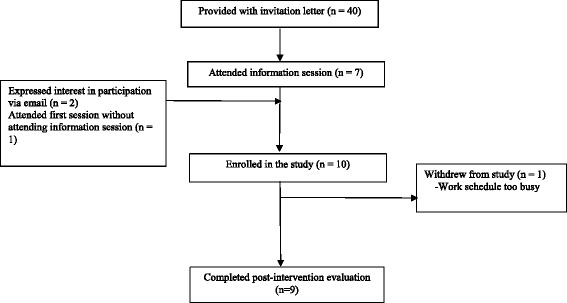
Study Flow Diagram

During the formative assessment stage of our research, one of the workplaces interviewed expressed interest in running a feasibility trial of PWT and one of its plants was chosen as the pilot site. The company is a large manufacturing company located in Guelph, Ontario. Employees of the plant work shift work and the plant is primarily composed of male employees (81 % male). From January to March 2015, we conducted a feasibility trial of PWT. Employees were eligible to participate if they were 1) over the age of 18 years, 2) had a child(ren) between the ages of 2–7 years, 3) were working the day shift, and 4) were literate in English. To recruit participants, the divisional HR department did an initial screen to determine those employees that met the eligibility criteria. All eligible employees (*n* = 40) were sent an invitation letter from HR inviting them to attend an on-site information session about PWT held at the same time and location as when the program was to be held. We recruited 10 participants for the program; however, after the second week. One participant had to withdraw from the study as his work schedule wouldn’t allow him to attend the weekly sessions (see Fig. 1 for the study flow diagram).

We present parent demographics in Table 2.

**Table 2:** Participant demographic characteristics

	*N* (%)
Relation to child	
Mother	4 (44.4 %)
Father	5 (55.6 %)
Marital status	
Married	6 (66.7 %)
Single, never married	2 (22.2 %)
Divorced	1 (11.1 %)
Race/Ethnicity	
White	5 (55.6 %)
Black	1 (11.1 %)
Chinese	1 (11.1 %)
Latin American	1 (11.1 %)
Southeast Asian	1 (11.1 %)
Total household income	
$20,000- $59,999	4 (44.4 %)
$60,000-$99,999	3 (33.3 %)
$100,000 or more	2 (22.2 %)
Education obtained	
Some high school	1 (11.1 %)
Graduated high school	5 (55.6 %)
Some college or technical school	1 (11.1 %)
College graduate	1 (11.1 %)
University graduate	1 (11.1 %)
Job category	
Laborer	6 (66.7 %)
Supervisory role	3 (33.3 %)
Born in Canada	
No	6 (66.7 %)
Yes	3 (33.3 %)

Of the nine participants who remained in the study, 5 were fathers (55.6 %) and 4 were mothers; just over half of participants self-identified as Caucasian (55.6 %) with the remaining participants coming from a variety of ethnic backgrounds. Most participants were working as labourers at the plant (66.7 %); the remaining participants had a supervisory role.

### PWT intervention

We present an overview of the program content in Table 3.

**Table 3:** Overview of the general parenting and weight-related topics addressed in Parents Working Together

Session	General parenting topic addressed	Weight-related topic addressed
1	Child–centered time	Being physically active with your child
2	Importance of family routines	Sleep: Creating a bedtime routine
3	Using praise and rewards	Reducing intake of sugar sweetened beverages
4	Setting limits	TV: Setting limits on TV
5	Threats and consequences & Using ignore and distract strategies	When not to use threats: Identifying your child’s hunger and satiety cues & Alternatives to food as reward
6	Problem solving with adults	Problem solving with partners and other caregivers about child’s health behaviours
7	Stress management/Work-life balance	Using physical activity to help manage stress

A member of the research team (LW) led the program sessions and a second research staff member attended all sessions to assess fidelity. As described above, PWT was adapted from PTT. PTT was adapted from a general parenting program, the Chicago Parent Program [37] to include content related to health behaviours – a more detailed description of PTT’s adaptation can be found elsewhere [18]. As was done in PTT, each PWT session included one primary parenting topic and one primary health behaviour topic. Content was presented through video vignettes and discussion questions meant to stimulate discussion amongst participants. As discussed previously, to be suitable for worksite delivery, we condensed the PWT sessions to 30 min in length to fit into the employees’ lunch time and the program was shortened from 9 to 7 sessions delivered over 7 weeks. To maintain the overarching goal of obesity prevention, all content related to health behaviours was included in PWT. To shorten the program, the focus of program adaptation was on condensing the parenting-related content. To do this, parenting topics that were addressed with multiple video clips were reduced to one or two video clips and some examples of “what not to do” were omitted (e.g., using labeled praise was included, but using unlabeled praise was omitted; using clear commands was included, but using begging, critical or unnecessary commands was omitted). Additionally, the two sessions on “threats and consequences” and “using ignore and distract strategies” were collapsed into a single session. Given that PWT aimed to alleviate stress at home that may influence job performance, specific discussion questions related to balancing work-life commitments were added to session 7 (Session 7: Managing Your Stress). PWT included a goal setting assignment each week (e.g., parents were encouraged to set their own parenting goal(s) and health behaviour goal(s) for the week). Participants were also sent home each week with a handout summarizing the important points from the session to serve as a reminder for participants as well as to help communicate the program messages to a spouse/other caregivers.

### Data collection

Given that this was a feasibility trial, program evaluation focused on process measures. To assess feasibility of the program, research staff monitored participant attendance. Research staff also assessed fidelity to the program facilitator’s manual by following along with sessions and noting any content that did not get covered/handouts that were not distributed during program sessions. To assess acceptability, attrition over the study period was measured. To assess participants’ satisfaction, we asked program participants via our final process survey to rate how satisfied they were with the overall program using a 4-point Likert scale (response options: very dissatisfied, dissatisfied, satisfied, very satisfied) as well as how satisfied they were with individual program components (e.g., the goal setting assignment, the videotaped examples the length of the sessions). They were also asked whether they would recommend the program to a co-worker, which was assessed using a 3-point Likert scale (response options: would not recommend, recommend, highly recommend). Parents were also asked to self-report their perceived change in confidence in handling their children’s health behaviours (e.g., “knowing when your child is full,” “limiting your child’s sugary beverage intake,” “helping your child engage in regular physical activity,” “limiting your child’s screen time to 1–2 h/day,” and “following a bedtime routine with your child”). Responses were provided on a 4-point Likert scale (response options: less confident than before, about the same as before, a little more confident, much more confident.)

As a secondary objective, we also assessed the impact of the program on parent and child weight-related behaviours as well as measures of general parenting strategies, parenting self-efficacy, feeding behaviours, and work-family conflict. Parent and child sugary beverage intake was assessed using questions from food frequency questionnaires previously validated in a pediatric populating (Native American and Caucasian children ages 1–5 years) [38, 39]. Average daily sleep for both parent and child was measured by self-reported sleep and wake times (assessed separately for weekdays and weekends). Parents were asked to report for themselves and their children, on average, the number of hours per day of TV/videos/DVDs watched in the past month (assessed separately for weekdays and weekends) [40]. Two items were used to assess child physical activity. Parents were asked to report on the length of time per day their child spends engaging in active play, as well as time engaged in outdoor play [41, 42]. Answers to these questions were rated on a 6-point Likert scale with response options ranging from 0 min to 2 h or more per day. Items from the Child Feeding Questionnaire (CFQ) were used to assess parental use of restriction and pressure to eat [43]. The CFQ has been validated for use by parents of children ages 2–11 years and grades responses a 4-point Likert scale (response options: strongly disagree, disagree, agree, strongly agree) [43]. Additionally, overall child nutritional risk was measured using the Nutrition Screen Tool for Every Preschooler (NutriSTEP), which is valid and reliable for use in preschoolers [44]. We used items from the Parenting Questionnaire (PQ) to assess general parenting strategies - specifically warmth and following through on discipline, measured on a 5-point Likert scale (response options range from never to very often) [45]. The PQ has been used among parents of preschool aged children [37]. General stress was measured using a parent stressor index that assessed stressors related to physical and mental health (an ordinal scale from 0 to 3 created by using the sum of the 2 domains: physical health (2 questions - “Would you say that your health, in general is excellent/good or fair/poor [excellent/good = 0 and fair/poor = 1] and “Do you have a health problem or condition that requires medical treatment or hospitalization on a regular basis?” [no = 0 and yes = 1]) and mental health (1 question – “Have you ever been diagnosed with any mental health condition, including clinical depression, anxiety disorder, or bipolar disorder?” [no = 0 and yes = 1]) [46]. Additionally, parent perceived stress was assessed using a single item from the 2006 Community Health Database [46]. Items from the Toddler Care Questionnaire were used to assess parenting self-efficacy [47]. This tool has been validated for use in toddlers and responses were provided on a 4-point Likert scale (Response options: not at all confident, a little bit confident, confident, very confident) [47]. To ensure that we adequately assessed work-life balance, as this was seen as important to employers, we included the Work-Family Conflict Scale in our measurement protocol which has been previously validated in a group of Master’s of Business Administration (MBA) graduates [48]. Responses are measured on a 5-point Likert scale with response options ranging from strongly disagree to strongly agree [48]. Parents were provided with a $10 gift card for completing the pre- and post-evaluation surveys.

Baseline surveys were provided to employees at the initial recruitment session and employees were asked to return the surveys the following week at week 1 of the program. Final surveys were provided at the end of week 7 and employees returned the surveys to the program facilitator (LW) 1 week later. It was expected that surveys would take approximately 20 min to complete.

### Data analysis

All data were analyzed using SPSS Version 22. For the process evaluation, frequencies were calculated from the participant process surveys. Additionally LW reviewed answers to open ended questions to categorize specific feedback from parents. For impact evaluation, paired samples t-tests were used to examine the change in outcome variables from pre- and post-intervention.

## Results: Feasibility Trial of Parents Working Together (PWT)

### Process results

Of the 9 parents who participated in the program, 89 % of participants attended 5 or more sessions with 44 % attending all 7 sessions. From the process survey results, 100 % of participants were either “satisfied” or “very satisfied” with the program and 100 % would recommend the program to a co-worker. 67 % of participants felt that the concerns that made them want to attend the program in the first place were satisfied and 78 % reported feeling either “a little more confident” or “much more confident” in handling their child’s behaviour at home (Table 4).

**Table 4:** Parents’ perceived confidence in managing their child’s behaviours

Confidence in:			
	About the same as before *N* (%)	A little more confident *N* (%)	Much more confident *N* (%)
Managing your child’s behavior at home	2 (22.2)	6 (66.7)	1 (11.1)
Knowing when your child is full	5 (55.6)	1 (11.1)	3 (33.3)
Limiting your child’s sugary beverage intake	3 (33.3)	4 (44.4)	2 (22.2)
Helping your child engage in physical activity	5 (55.6)	3 (33.3)	1 (11.1)
Limiting your child’s screen time	4 (44.4)	2 (22.2)	3 (33.3)
Following a bedtime routine with your child	5 (55.6)	1 (11.1)	3 (33.3)

Despite enjoying the program, participants had suggestions for improvement. Their primary criticism of the program was that the sessions were too short; participants suggested an ideal length to be 45 min to 1 h in duration with a specific focus on including more time for discussion.

### Preliminary impact results

Although changes in children’s weight-related behaviors, (i.e., sleep, TV watching, active play, and sugar-sweetened beverage intake), appeared to be in the desired direction at follow-up, the changes were not statistically significant (Table 5). Parents reported that their children consumed fewer servings of sugar sweetened beverages at post-intervention than at baseline (change = -0.3 servings/day, *p* = 0.60), and watched fewer hours of TV (change = -0.1 h/day spent watching, *p* = 0.43). Parents also reported an increase in child sleep duration from baseline to post-intervention (change = 0.2 h/day spent asleep, *p* = 0.15) as well as an increase in child physical activity in terms of both active play and outdoor play (change = 5.7 min/day spend in active play, *p* = 0.60; change = 8.9 min/day spend in outdoor play, *p* = 0.55).

**Table 5:** Change in parent and child outcomes from baseline to post intervention for participants in the Parents Working Together intervention

Outcome	Baseline mean (SD)	Post intervention mean (SD)	Change (95 % CI)	*p*-value
Parent outcomes				
Parenting self-efficacy Range of possible scores: 22–88	73.1 (5.4)	77.7 (7.0)	4.6 (-1.5,10.7)	0.12
General parenting strategies				
Range of possible scores: 1–5				
Warmth	3.7 (0.5)	3.9 (0.4)	0.2 (-0.1,0.5)	0.19
Follow through with discipline	2.4 (0.7)	2.3 (0.8)	-0.1 (-0.5,0.4)	0.73
General stress	0.3 (0.5)	0.0 (0.0)	-0.3 (-0.6,0.1)	0.17
Range of possible scores: 0–3				
Self-reported Stress	7.1 (2.7)	5.7 (3.2)	-1.4 (-4.2,1.4)	0.28
Range of possible scores: 1–10				
Feeding behaviours				
Range of possible scores: 1–4				
Food as reward	2.3 (0.5)	2.4 (0.5)	0.1 (-0.2,0.4)	0.35
Food restriction	2.7 (0.6)	3.0 (0.5)	0.3 (0.7,0.5)	0.02
Pressure to eat	2.6 (0.5)	2.4 (0.7)	-0.2 (-0.7,0.3)	0.37
Work-family conflict				
Range of possible scores: 1–5				
Work interferes with family	2.4 (0.5)	2.3 (1.3)	-0.1 (-1.3,1.1)	0.86
Family interferes with work	2.4 (0.3)	1.8 (1.2)	-0.6 (-1.6,0.3)	0.16
Sugar sweetened beverage intake (servings/day)	1.0 (1.1)	0.7 (0.4)	-0.3 (-1.4,0.6)	0.41
TV duration (hours/day)	2.3 (1.0)	2.1 (0.8)	-0.1 (-0.8,0.5)	0.67
Sleep duration (hours/day)	7.5 (0.7)	7.4 (1.2)	-0.1 (-0.8,0.7)	0.84
Child outcomes				
Sugar sweetened beverage intake (servings/day)	1.3 (1.4)	1.0 (0.9)	-0.3 (-1.6,1.0)	0.60
TV duration (hours/day)	2.3 (0.8)	2.2 (0.7)	-0.1 (-0.3,0.2)	0.43
Sleep duration (hours/day)	10.8 (0.5)	11.0 (0.6)	0.2 (-0.1,0.5)	0.15
Active play (minutes/day)	84.1 (33.0)	89.8 (27.4)	5.7 (-18.2, 29.5)	0.60
Outdoor play (minutes/day)	83.6 (39.7)	92.5 (35.1)	8.9 (-27.0, 44.8)	0.55

In terms of parent weight-related behaviours, parents reported a slight decrease in sugar sweetened beverage consumption; however, this decrease was not statistically significant (change = -0.3 servings/day, *p* = 0.41). Sleep duration and TV watching remaining relatively unchanged from baseline to post-intervention (Table 5). Parents’ use of restrictive feeding practices increased from baseline to post-intervention (restriction change score = 0.3, *p* = 0.02).

Changes in general parenting strategies were in the desired direction at post-intervention; however, the changes were not statistically significant (Table 5).

Parenting self-efficacy increased from baseline to post-intervention (self-efficacy change score = 4.6, *p* = 0.12). As well, there was a decrease in self-reported general stress (self-reported general stress change score = -1.4, *p* = 0.28). Parents reported a decrease in both work interfering with family and family interfering with work from baseline to post-intervention. Although confidence intervals substantially overlapped zero for both measures, it is worth noting that the change in family interfering with work was of a greater magnitude than work interfering with family (work interferes with family change score = -0.1, *p* = 0.86; family interferes with work change score = -0.6, *p* = 0.16).

## Discussion

Results from this study indicate that a workplace parenting program that addresses general parenting and health behaviour messages is of interest to employers and possibly worth implementing. In addition to expressing general support for a workplace parenting program, workplace representatives also identified some key recommendations and logistical considerations that would maximize the success of the intervention. These included: keeping the number of program sessions offered to the minimum required, holding sessions in the workplace during workday hours, using a traditional lunch and learn format, and shortening program sessions to fit into the employees’ allotted lunch break.

Using these learnings we adapted Parents and Tots Together (PTT), a general parenting program with embedded weight-related messaging, to be delivered in the workplace as the Parents Working Together (PWT) program. The main finding from the feasibility trial of PWT was that the program was both feasible to implement and acceptable to employees of a large manufacturing company in Southwestern Ontario. Attendance rates were high with 89 % attending most of the sessions offered. PWT was also well received by parents and 100 % of participants stated they would recommend the program to co-workers. Despite good attendance and a high level of participant satisfaction there were suggestions for improvement of the program including allowing more time for discussion and lengthening sessions.

PWT fits into the body of literature on workplace parenting programs, which suggests that, when parenting programs are delivered in parents’ workplaces, attendance is high and attrition is low [26–32]. A novel finding of PWT is that a worksite parent program focused on general parenting and weight-related messages can be successful at recruiting fathers to the intervention; 55.6 % of the participants in PWT were fathers. Parenting programs are typically attended primarily by mothers both in the workplace [27, 30, 31] and in community settings [18, 20, 21, 37]. Only Let’s Talk! an HIV prevention parenting program was successful at recruiting a comparably high percentage of fathers (65 %) to their program [32]. As with PWT, this success in father recruitment may largely be due to the types of workplaces targeted. Similar to the worksite used in our trial, the worksites in Let’s Talk! included 5 City departments (Solid Waste, Roads and Stormwater, Municipal Libraries, Electricity Maintenance, and Parks and Recreation), which likely consist of a large proportion of male employees. It may be that targeting male-dominated workplaces could be an effective way to engage fathers in childhood obesity prevention interventions. Engaging fathers in obesity prevention initiatives is critical given their important and unique role in the development of healthy weights in their children [49–51].

Results from the impact evaluation of our feasibility trial suggest that PWT may be effective in changing weight-related behaviours for both parents and children in the desired direction as well as improving parenting self-efficacy, decreasing parent-reported general stress, and improving measures of work-life balance. Although we saw non-significant changes in the desired direction for both the weight-related behaviours and parenting outcomes, the magnitude of change in the parenting outcomes was quite small. To shorten the program, we decided to minimize the parenting content and focus on the weight-related content. It may be that the parenting-related messaging was cut back too much and the dose was insufficient to see substantial change in the desired direction. In their 14-session parenting intervention, ParentCorps, [52] demonstrated significant improvements in parent knowledge (change = 0.93, *p* < 0.001) and use of effective parenting practices (change = 0.12, *p* = 0.013), as well as significant decreases in child problem behaviours (change = -1.67, *p* = 0.022), suggesting that an increased dose of general parenting content may lead to improved outcomes in this area.

Contrary to expectations, parents also reported a significant increase in restrictive feeding practices (i.e., restriction of types/amounts of foods provided to children). Although PWT does not promote restriction, it is possible that once made aware of the importance of healthful eating and reducing the intake of sugar sweetened beverages, parents may feel that restriction is needed; in particular in an obesogenic environment that promotes unhealthful dietary intake. The research to date on restriction has been mixed, with some studies showing that the use of restrictive feeding practices is harmful and increases a child’s obesity risk [11, 53, 54] and others finding the opposite [55]. More research is needed to inform evidence-based guidance on feeding practices, in particular related to use of restriction.

Future trials of PWT should focus on ways to include more time for discussion since we heard from our participants that this was missing from program sessions. One option may be to include an online component – either an online discussion board or a reverse classroom scenario where participants review the content and watch the video vignettes at home online and then program sessions could focus on discussion. The current study only included parents with children aged 2–7 years; future trials should explore whether PWT can be adapted to include content that is relevant to parents with children of a wider age range, which would allow more employees to participate.

A primary strength of this study is that it utilized a mixed methods sequential design whereby a thorough formative assessment was used to inform the quantitative piece. Not only do formative assessments increase the likelihood for success of an intervention [56], they can also help identify key collaborators or champions for the resulting program. In our case we were able to promote our intervention and engage a worksite interested in piloting our program.

This study had several limitations. First, the sample for our feasibility trial was small, which limits our power to detect statistical significant changes in our secondary outcomes (i.e., parent and child behaviours). However, the effect sizes that we did find for most measures were quite small indicating that more intensive intervention is needed for meaningful behaviour change. Second, our samples for both the formative assessment and pilot study came from one geographical area (i.e., southern Ontario), which may limit the generalizability of our findings. Third, we did not have a control group for our feasibility trial; therefore, it is unknown whether our results are due to participation in our program or something external to the program. Fourth, our measures were all self-reported by the parents for both themselves and for their children. This increases the likelihood of self-report bias and errors due to poor recall. Additionally, not all measures used have been validated for use in our specific population. A future randomized control trial should include tools validated for use with parents of children ages 2–7 and objective measures of activity and sleep behaviours. Finally, we did not include any long-term follow up with participants, therefore we are unsure whether the results we saw immediately post-intervention were sustained. Such information could be useful for determining whether we need to add any maintenance elements to our program.

## Conclusion

Results from the current study showed that a workplace program that addresses general parenting and health behaviour messaging resonates with employers and resulted in a number of suggestions from employer representatives on ways to structure the program to fit into the logistical constraints of a workplace setting. The main finding from the feasibility trial was that PWT is feasible and acceptable to employees. Our results suggest that workplaces should be considered as potential settings to hold interventions as they are convenient to parents and there is the potential to maximize attendance and minimize the rate of attrition, which are common problems with community-based parenting programs [24, 57]. A sufficiently powered, clustered-randomized trial is needed to test the impact of PWT on parent and child weight-related behaviours and outcomes.

## Additional file

Additional file 1: Categories and representative quotes from interviews with employers. The main categories from the formative assessment and representative quotes from the interviews with employers that represent these categories. (DOCX 126 kb)

## Abbreviations

PTT: Parents and Tots Together
PWT: Parents Working Together
HR: Human Resources
NutriSTEP: Nutrition Screen Tool for Every Preschooler
MBA: Master’s of Business Administration
