# Supplementary material for: Parents Working Together: development and feasibility trial of a workplace-based program for parents that incorporates general parenting and health behaviour messages
Source: BMC Public Health. 2016 Nov 10;16:1154. doi: 10.1186/s12889-016-3817-z (PMC5105296; doi:10.1186/s12889-016-3817-z)
Supplement: Additional file 1: — Categories and representative quotes from interviews with employers. The main categories from the formative assessment and representative quotes from the interviews with employers that represent these categories. (DOCX 126 kb) [file 12889_2016_3817_MOESM1_ESM.docx]

Additional file 1. Categories and representative quotes from interviews with employers

| **Theme** | **Representative quote from interview participants** |
| --- | --- |
| Work-Life Balance Issues Exist for Employees and there is Interest in a Workplace Parenting Program | - Employees will report to you that this is probably their number 1 struggle when it comes to working is finding...striking that right balance between their work life and their family life. [P001] - I think stress, you know, is certainly on the rise. We see it in our claims costs, we hear about it on the news, read it in the newspaper, you know mental health obviously being a huge issue right now that all the workplaces are tackling. And a lot of the mental health and issues in work are things related to balance and workload. [P010] - Work-life balance is very important to people’s wellness because people, in general, they don’t live to work, they work to live and family should always come first [P001] - So I’d have to back up a lot to really just talk about how I see the value of something like this for employees. Because I think employees are looking for that and I think employers are getting pushed more and more to provide the support to that. [P002] - Because, certainly, if it’s something I can do to assist the employees in improving their lifestyle or their work-life home and balance or their…just themselves, so they feel better, and it’s 30 minutes of free time on a volunteer basis and I’m just providing the room, why wouldn’t I? There’s no better scenario than that as an employer, as far as I’m concerned, right? [P004] - Something that would be worth piloting. [P004] |
| Existing or Past Program Strategies Used by Employers | - You know, flex hours, you know, coming in…as long as you’re working your 7.5-8 hour day, however you do it, depending on what your kids’ needs are, you can be flexible [P003] - And we do offer flex programming…or flex work arrangements when we can to accommodate that kind of thing. [P006] - We kicked off a work-life balance wellness series starting in September of 2013. And our goal was to take a look at the different dimensions of wellness and integrate lunch and learn seminars that hit on different areas of work-life balance, or that could improve work-life balance. [P011] - And we’ve got a really good EFA program, Employee Family Assistance program. So through that, they have parenting help, they have marital counseling, they have addictions, they have financial. Whatever an employee may need, it’s there. [P006] |
| Barriers to Program Implementation and Ways to Address Them | - We have a hard time right now with any of our learning and development programming getting attended, because of the demand on people’s time. [P003] - What draws them in is prizes, it sounds so shallow but it’s true, prizes and food. And the hope is by having those, you get the people in there and you hope somebody walks away with [something]. [P009] - Everybody thinks they’ve discovered the next way to do something better and I think we’ve really complicated what’s very basic, right? So I think structured, focused sessions are the biggest bang for your buck. [P004] |
| Suggested Program Logistics and Strategies  Program Length  Session Length  Program Location and Format | - So it would truly be effective if you got the company’s commitment to do 4 sessions or, you know, 3 half day sessions, then people are going to be more committed to come to the whole thing. [P008] - You might not get people to attend every week for 9 weeks. You might find that people will want a shorter…like if you do 4 weeks at a time then you might get a higher commitment. 9 might be a little bit too much. You might get people show up to some but not to all. [P001] - I think in the workplace that might be a little bit too long. I was mentioning 30 to 45 minutes so that you can capture people’s attention and they can find that in their workday. But 90 minutes might be a little bit too long in the workplace. [P001] - You could do a 30 minute lunch and learn. I’m sure [P004] - There’s a level of trust that comes with it. And all these programs, sometimes just an employer endorsing it is enough for people to go “oh OK, this is good. If they, my HR manager and GM support this, it must be meaningful.” [P004] - It’s on-site, it’s accessible, I’m already here [P004] - No I think that’s convenient. People love that convenience factor. I think that’s a bonus to have it in the workplace. [P001] - …on company time, which we all know is the greatest turnout and participation…because I don’t know about you, but like me, when I’m done my day, the last thing I want to do is stay longer, even if it’s free. I’m tired. [P004] |
| Suggested Program Evaluation | - Anything that…I believe, anything that can be drawn to the bottom line of that company. So anything that the CFO will listen to and believe in. So if it’s reduced sick days, if it’s reduced turnover for people with young children [P008] - No I think that the participation rates and the completion rates are good. Umm I’d always be…I’m always interested in any comments that people make about what worked and what didn’t work…As an employer. I’d be curious as a person. As an employer, I would just be looking at participation and satisfaction with the program. [P007] |
| Suggestions for Marketing the Program  Marketing to Employees    Marketing to Employers | - It would be more focused so that it would be like, you know what, this is a normal challenge, or this is a normal feeling. People want to know that, you know, when their kid acts up and they freak out at them and yell once, that other people experience that and that it doesn’t make them an awful parent. So marketing it around, you’re not alone, let us help you. [P009] - You could run it very positively though. [P004] - I think that the managers and the supervisors would be most interested in supporting something like this for their employee if it is going to remedy a difficult situation that they’re already having. [P003] |
